# Sudden sensorineural hearing loss in a post‐COVID‐19 patient

**DOI:** 10.1002/ccr3.4956

**Published:** 2021-10-17

**Authors:** Santoshi Pokharel, Sumita Tamang, Shankar Pokharel, Rajeev Kumar Mahaseth

**Affiliations:** ^1^ College of Medicine Nepalese Army Institute of Health Sciences Kathmandu Nepal; ^2^ HAMS Hospital Kathmandu Nepal; ^3^ Department of Otorhinolaryngology Shree Birendra Hospital Kathmandu Nepal

**Keywords:** coronavirus, COVID‐19, SARS‐CoV‐2 RT‐PCR, Sudden Sensorineural Hearing Loss (SSNHL)

## Abstract

A detailed history and diagnostic evaluation for recent or past COVID‐19 infection is vital in patients presenting with Sudden Sensorineural Hearing Loss (SSNHL) since SSNHL could be a sequelae of COVID‐19 and timely diagnosis and intervention could significantly improve hearing and quality of life.

## Graphical abstract

Diagnostic evaluation of COVID‐19 should be taken into consideration for patients presenting with SSNHL in this COVID‐19 pandemic. An extensive research is needed to understand the association and onset of SSNHL in COVID‐19.

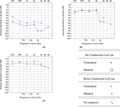

## 1 INTRODUCTION

Sudden Sensorineural Hearing Loss is defined as sensorineural hearing loss of 30dB or greater over at least three contiguous audiometric frequencies occurring within a 72‐h period,^1^ usually accompanied by tinnitus or temporary spells of vertigo. It has an annual incidence of about 11‐77/1,00,000 cases in United States^2^ and mostly occurs in 65 years and older age group with a male predominance of 1.07:1. Hearing loss can be complete or partial but mostly unilateral. Despite being a commonly encountered phenomenon, most often the exact cause of SSNHL remains obscure and is termed as idiopathic in origin. Other causes that may possibly lead to deafness can be infectious, autoimmune, traumatic (head injury, ear operation), otologic (Meniere's disease), malignancies (acoustic neuroma, schwannoma), vascular (thrombotic, embolic) in origin. Most often viral illness (Cytomegalovirus, Herpes virus) leads to hearing loss with no definite mechanism. Since severe acute respiratory syndrome coronavirus 2 (SARS‐CoV‐2) is a viral infection, a few cases around the globe have been reported correlating SSNHL with COVID‐19. So SSNHL could be a sequelae of COVID‐19, provided that other etiologies have been ruled out in a previously normal healthy hearing person.

## 2 CASE PRESENTATION

**FIGURE 1:**
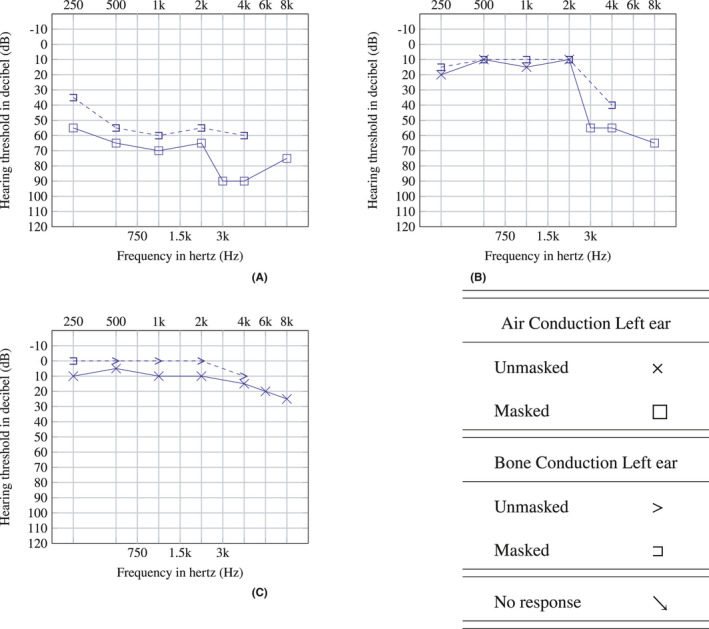
Pure Tone Audiometry (A) at the time of presentation (B) after initiation of oral steroid (C) at completion of oral steroid

**FIGURE 2:**
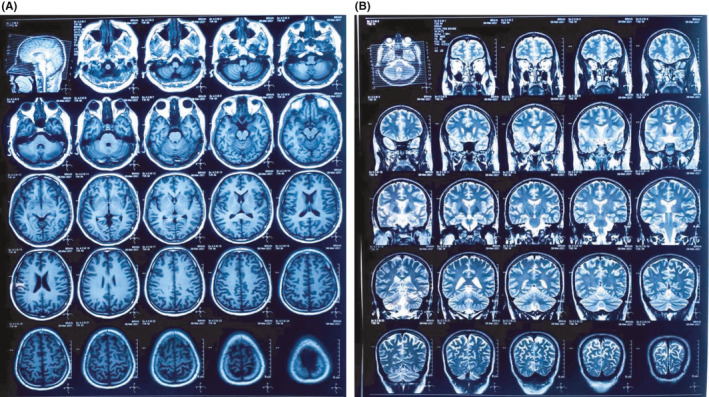
The MRI scan of brain

We present a case of a 27‐year‐old Nepalese male patient, apparently healthy with no known comorbidities or any ear pathology. He initially had malaise and fatigue for 3–4 days. Later he developed loss of smell and taste sensation for which he was suggested RT‐PCR test for SARS‐CoV‐2 and was tested positive. His vital parameters and saturation were normal at room air, so he was advised for home isolation. His home isolation period was uneventful except for mild symptoms. After 1 month, all symptoms resolved but he developed a ringing sensation followed by acute onset of complete hearing loss in the left ear. He made a visit to a nearby hospital on the same day and a follow–up visit to the same hospital after 3 days, and audiometric investigations were done at each visit and oral steroids were prescribed. He then had three subsequent visits to our hospital where workup for hearing loss was done along with continuing oral steroids. Physical examination detected no structural abnormality. Otoscopic examination revealed normal external auditory canal. General screening test was done by whispering test, which showed decreased response from the affected left ear. Tunning fork test with 512Hz showed positive Rinne's test on both ears and Weber test was lateralized to unaffected ear. Pure Tone Audiometry (PTA) was done which showed moderately severe SSNHL on the left side (Figure 1A) with normal hearing limit on the right side. Repeat PTA tests were done on day 7 and day 14 after the administration of oral steroid, which showed the significant improvement in hearing as shown in (B) and (C), respectively, of Figure 1. There was no evidence supporting other causes for left‐sided SSNHL as there was no history of ear discharge, trauma, use of ototoxic drugs during his isolation period and no exposure to loud noise. The MRI of brain showed normal scan (Figure 2). Patient was managed by oral steroids on tapering dose. With timely use of the steroids, his hearing improved significantly.

## 3 MANAGEMENT

The main treatment the patient received for his symptoms was the administration of oral steroids in tapering dose, which resulted in significant improvement in his hearing as seen in PTA (Figure 1). No clear etiology for his SSNHL was established from history, examination, and investigations, but a timely hospital visit and timely administration of medication improved his hearing significantly.

## 4 DISCUSSION

Sudden Sensorineural Hearing Loss is a medical emergency which necessitates immediate attention. With the annual incidence of 11–77 per 1,00,000 cases in United States,^2^ it is a relatively common complaint in otologic and audiologic practices. The causes of SSNHL are speculative and often multifactorial. In majority of cases, the exact pathology is not confirmed so that SSNHL is termed as idiopathic. Other postulated factors contributing to hearing loss could be infectious, autoimmune, traumatic, otologic, carcinomatous, and vascular (thrombotic/embolic) in origin.^3^ SSNHL is supposed to occur following viral infection. Several postulated mechanisms of viral infection leading to hearing loss are by damaging inner ear structures, including inner hair cells and organ of Corti, and by induction of host immune‐mediated damage.^4^ Also, cardiovascular and virus induced coagulopathy and inflammatory edema can lead to cochlear ischemia and subsequent hearing loss.^3^ Apart from other viral infections, SSNHL in COVID‐19–infected person is being reported from different parts of the world. The first case of hearing loss in a COVID‐19 positive patient was reported on March 15, 2020, from Thailand.^5^ The case that we are reporting in this article is a 27‐year‐old Nepalese male patient with no known prior medical conditions presenting with sole complaint of tinnitus and acute onset of left‐sided hearing loss after 30 days of COVID‐19 infection. We applied the criteria of Satar et al^6^ before proposing the relationship between SARS‐CoV‐2 infection and occurrence of SSNHL in our case as follows:

1. The case was laboratory confirmed by RT‐PCR for SARS‐CoV‐2 infection.
2. Hearing loss is documented during the downward phase of infection that is around after 4 weeks, which could be possible because of antigen‐antibody complex or immune reaction to viral infection.
3. Signs for vestibular involvement were ruled out although our patient did not complaint of dizziness, vertigo, or no focal signs of neural involvement.
4. Other causes of hearing loss were ruled out as there was no history of acoustic trauma, exposure to loud noise, use of ototoxic medication during isolation period or before the period of COVID‐19 infection, no history of prior otologic problems. Retrocochlear lesions such as vestibular schwannoma, multiple sclerosis were ruled out via MR imaging of brain.

To correlate our findings, we did a detailed literature search and found out that though plenty of papers mentioned about SSNHL, there are only few papers correlating SSNHL and COVID‐19 and appropriate management of SSNHL. In a comparative study by Mustafa et al,^7^ the amplitudes of transient evoked otoacoustic emissions (TEOAEs) and thresholds of pure‐tone audiometry were significantly worse in COVID‐19–infected patients in comparison to normal non‐infected subjects concluding COVID‐19 infection could have deleterious effects on cochlear hair cell functions despite being asymptomatic.^7^ 1 out of 5 patients tested positive for SARS‐CoV‐2 infection presenting with sole complaint of unilateral SSNHL in otorhinolaryngology outpatient clinic.^8^ 53‐year‐old Turkish male patient reported complete unilateral SSNHL following COVID‐19 infection and was managed with steroids.^9^ A case report from India mentioned a 49‐year‐old diabetic male patient with hearing loss after 3 months of COVID‐19 infection.^10^ Acute‐onset hearing loss is noted in a young COVID‐19 patient who denied prior otologic problems and use of ototoxic medications.^11^ Rhman et al discussed a case of 52‐year‐old man presented with tinnitus and acute‐onset left–sided hearing loss after COVID‐19 infection and managed with intratympanic steroids which improved his hearing.^12^

Exact etiopathogenesis of SSNHL in COVID‐19 patients is not well known. SARS‐CoV‐2 is believed to bind to angiotensin‐converting enzyme 2 (ACE2) receptors,^13^ which is present on alveolar epithelial cells, endothelial cells, and several other organ systems. Varga et al detected SARS‐CoV‐2 elements in endothelial cells, with evidence for the induction of endothelial dysfunction and cell apoptosis.^14^ As hypothesized by Harenberg et al, hearing loss in COVID‐19 patients could be a result of endothelial dysfunction with micro‐thrombosis at the level of auditory center in temporal lobe, the auditory nerve or the cochlea.^15^ Viral infection of olfactory nervous system is believed to contribute to the pathophysiology of COVID‐anosmia, in a similar way, viral infection of auditory nervous system could induce symptoms of hearing loss.^16^ Apart from this, SARS‐CoV‐2 is believed to cause an inflammatory response and increase in cytokines such as IL‐1, IL‐6, and TNF alpha,^17^ which causes a direct entry into cochlea and causes inflammation leading to cellular stress response leading to SSNHL. Degen et al reported a case of 60‐year‐old man with COVID‐19 pneumonia who developed complete deafness on right ear and profound SSNHL on left side with MRI findings suggesting the signs of inflammatory process in cochlea, which could have been virus triggered, immune‐mediated inflammation in cochlea.^18^ Coagulopathic disorder with thrombotic events in COVID‐19 patients is described which can lead to SSNHL.^19^ Neurotropic and neuroinavsive property not found in MERS and SARS has been documented in Corona Virus.^20^, ^21^ Furthermore, histological reports of patients with SSNHL have shown the loss of hair cells and supporting cells of Organ of Corti without inflammatory infiltrate, suggesting the pathology of idiopathic SSNHL.^22^ Mastoid specimens tested RT‐PCR positive for SARS‐CoV‐2 in a post mortem bilateral cortical mastoidectomy performed in a post‐COVID‐19 patient^23^ suggesting the presence of virus in middle ear and mastoid.

## 5 CONCLUSION

No clear etiology for SSNHL was found in our case and it was speculated that this could be due to COVID‐19. As there is no obvious pathophysiologic mechanism associating COVID‐19 with SSNHL, the association remains obscure. The temporal concordance between SARS‐CoV‐2 infection and onset of SSNHL could not be clarified.

## CONFLICT OF INTEREST

None declared.

## AUTHOR CONTRIBUTIONS

Santoshi Pokharel wrote the manuscript and contributed in conceptualizing, writing and reviewing. Sumita Tamang and Shankar Pokharel contributed in data collection, writing and reviewing. Rajeev Kumar Mahaseth contributed in reviewing and supervision.

## ETHICAL APPROVAL

This case was written and published with the consent of the patient.

## Data Availability

Data available on request from the authors.
